# Ultra‐fast MRI for dementia diagnosis and treatment eligibility: A prospective study

**DOI:** 10.1002/alz.70341

**Published:** 2025-06-11

**Authors:** Miguel Rosa‐Grilo, Haroon R. Chughtai, David L. Thomas, Daniel C. Alexander, Millie Beament, Christopher R. S. Belder, H. Rolf Jäger, Emma A. Lim, Nicholas Magill, Dermot Mallon, Jennifer M. Nicholas, Owen Nicholas, Floey Urban, Geoff J. M. Parker, Frederik Barkhof, Catherine J. Mummery, Nick C. Fox

**Affiliations:** ^1^ Dementia Research Centre UCL Queen Square Institute of Neurology University College London London UK; ^2^ UCL Hawkes Institute University College London London UK; ^3^ Department of Medical Physics and Biomedical Engineering University College London Malet Place Engineering Building London UK; ^4^ Advanced Research Computing (ARC) Centre University College London London UK; ^5^ Neuroradiological Academic Unit Department of Brain Repair and Rehabilitation UCL Queen Square Institute of Neurology University College London London UK; ^6^ Department of Computer Science University College London London UK; ^7^ Neurology Department Royal Adelaide Hospital and Queen Elizabeth Hospital Adelaide Australia; ^8^ Adelaide Medical School The University of Adelaide Adelaide Australia; ^9^ Department of Imaging Imperial College Healthcare NHS Trust London UK; ^10^ Lysholm Department of Neuroradiology National Hospital for Neurology and Neurosurgery, Queen Square London UK; ^11^ Department of Medical Statistics London School of Hygiene & Tropical Medicine London UK; ^12^ Department of Statistical Science University College London London UK; ^13^ Bioxydyn Limited, St James Tower Manchester UK; ^14^ Department of Radiology and Nuclear Medicine Amsterdam UMC, Vrije Universiteit Amsterdam The Netherlands

**Keywords:** advanced parallel imaging, Alzheimer's disease, dementia diagnosis, eligibility for disease‐modifying therapies in Alzheimer's disease, MRI acceleration techniques, structural MRI, wave‐CAIPI

## Abstract

**INTRODUCTION:**

Magnetic resonance imaging (MRI) is crucial for dementia diagnosis and a pre‐requisite for amyloid‐lowering therapies in Alzheimer's disease. Despite guidelines, many patients never undergo MRI due to limited scanner availability. Shorter scan times would reduce costs and patient burden. We developed and tested a fast MRI protocol incorporating highly accelerated sequences.

**METHODS:**

We compared blinded neuroradiologist assessments of a fast protocol with the standard‐of‐care protocol in a prospective real‐world study. We estimated agreement coefficients to evaluate reliability.

**RESULTS:**

The fast protocol cut scan times by 63% and showed non‐inferior reliability measures for diagnosis, visual scale ratings, and disease‐modifying therapy eligibility assessment. Between scan‐type, intra‐rater reliability for diagnosis was greater than inter‐rater reliability on the standard‐of‐care protocol (ratio of 1.37, 95% confidence interval: 1.21–1.58).

**DISCUSSION:**

This study proposed and applied a way of showing non‐inferiority of a highly accelerated dementia protocol. Ultra‐fast protocols could improve MRI access and patient equity and support the implementation of disease‐modifying therapies.

**Highlights:**

- The fast dementia protocol with four core sequences reduced acquisition time by 63%.
- The fast scan showed non‐inferior reliability for diagnosis and visual ratings.
- Assessment for disease‐modifying therapy eligibility was similar between scan types.
- Fast protocols may improve access to magnetic resonance imaging and diagnosis in dementia.

## 1 BACKGROUND

The growing prevalence of dementia as populations age,^1^ and the challenge of delivering disease‐modifying therapies (DMTs) for Alzheimer's disease (AD),^2^ means health care systems are under unprecedented pressure to deliver a timely diagnosis. Structural brain imaging plays a critical role in diagnosis,^3^ with the choice between computed tomography (CT) and magnetic resonance imaging (MRI) depending on various factors including scanner availability, scan duration, cost, and patient cooperation.^4^, ^5^, ^6^ MRI is favored for its excellent soft tissue contrast, lack of ionizing radiation, and positive predictive diagnostic value.^7^ MRI is also a pre‐requisite for amyloid‐lowering DMT eligibility in view of its utility not only for diagnosis but also for detecting safety signals such as focal edema or cerebral microhemorrhages.^8^, ^9^

Notwithstanding its advantages, MRI is constrained by availability and relatively long acquisition times. Lengthy sequences can make it more challenging for individuals to remain still, potentially resulting in movement artifact or an inability to tolerate the scan. Shorter (accelerated) scans could therefore increase MRI capacity, reduce cost and patient burden, and improve access to diagnosis and treatments.

Typically, MRI dementia protocols require at least 20 min to acquire.^10^ Beyond the traditional goal of excluding surgically treatable pathology—which accounts for a minority of cases—imaging aims to determine the extent and pattern of brain atrophy and assess cerebrovascular disease burden and markers of cerebral amyloid angiopathy.^11^ These protocols generally include T1‐weighted gradient echo (T1w), fluid‐attenuated inversion recovery (FLAIR), T2‐weighted turbo or fast spin echo (T2w), and T2*‐weighted gradient recalled‐echo or susceptibility‐weighting imaging (T2*w/SWI).^10^, ^12^ High‐resolution three‐dimensional (3D) T1w acquisitions have been favored for the assessment of brain atrophy.^13^ In addition, volumetric (3D) FLAIR and SWI sequences offer enhanced sensitivity for detecting cortical and subcortical vascular lesions and microhemorrhages.^14^, ^15^

In recent years, substantial efforts have been made to accelerate the acquisition of 3D MRI sequences.^16^, ^17^, ^18^ Wave‐controlled aliasing in parallel imaging (wave‐CAIPI) is an innovative advancement in parallel imaging that leverages a modified k‐space trajectory to enable higher acceleration.^19^, ^20^, ^21^ Wave‐CAIPI has been evaluated mostly in single‐sequence comparative studies.^22^, ^23^, ^24^, ^25^ Studies have focused on multiple intracranial pathologies or have had relatively small sample sizes of individuals with neurodegenerative disease.^22^, ^24^, ^26^ To our knowledge, the performance of a highly accelerated full dementia protocol for differential diagnosis has not been evaluated in a clinical setting for people with cognitive concerns.

In this study we investigate the diagnostic performance of an accelerated dementia protocol (fast scan) and compare it with the standard‐of‐care protocol (clinical scan). Critically, we evaluated scans of individuals referred to a real‐world outpatient cognitive service in whom brain imaging was being planned as part of the routine diagnostic workup. This outpatient setting includes individuals with various brain pathologies associated with cognitive decline (e.g., AD), as well as those without significant intracranial pathology but presenting with other contributing factors (e.g., mental health conditions or functional cognitive symptoms). We sought to determine whether the reliability of radiological assessments between the fast and clinical scans was non‐inferior to the reliability among different neuroradiologists interpreting the same clinical scans, thereby demonstrating that the fast scan performs comparably in a clinical setting.

## 2 METHODS

### 2.1 Study population

Recruitment for this prospective blinded comparison study took place between November 2022 and February 2024. We enrolled patients from the specialist cognitive disorders service at the National Hospital for Neurology and Neurosurgery, UK, who were undergoing an MRI brain scan as part of their routine diagnostic assessment—participants were only approached after their treating clinician had requested an MRI. There were no exclusion criteria apart from the standard requirements for MRI scanning,^27^ an age range of 50–90 years, and the capacity to give informed consent. Written informed consent was obtained for all participants. Demographic data were collected prior to scanning. Ethical approval was granted by the NHS Health Research Authority London (REC reference 21/LO/0815). The study was performed in accordance with the 1964 Declaration of Helsinki and its later amendments.

### 2.2 Image acquisition

Imaging was performed on a Siemens MAGNETOM Prisma Fit 3T system using a 64‐channel array receiver coil. Wave‐CAIPI sequences were provided by Siemens Healthineers (Erlangen, Germany) as Work‐In‐Progress packages.

All participants underwent imaging with the clinical and fast protocols in the same session. Sequences from these two protocols were acquired in an interleaved manner. The total acquisition times for these protocols were 17:39 and 6:29 (minutes:seconds), respectively. Scan parameters for the clinical and optimized fast protocols are shown in Table 1. Prior to this study, we optimized the fast sequence parameters on a separate group of patients and healthy volunteers.^28^, ^29^

**TABLE 1:** Acquisition parameters for clinical and fast protocols.

Sequence	Characteristic	Clinical protocol	Fast protocol
3D T1‐weighted (T1w)	Resolution	1.05 × 1.05 × 1.2 mm^3^	1.1 × 1.1 × 1.1 mm^3^
	Orientation	Sagittal	Sagittal
	Field of view	270 mm × 270 mm	280 mm × 280 mm
	Number of slices	176	192
	Parallel imaging	GRAPPA × 2	Wave‐CAIPI 3x2
	TI/TR/flip angle	900 ms/2300 ms/ 9°	800 ms/2650 ms/ 9°
	Scan time	5 min 12 s	1 min 34s/1 min 37 s (with fat‐saturation)*
Multislice 2D T2‐weighted (T2w)	Resolution	0.625 × 0.625 × 3 mm^3^	0.625 × 0.625 × 3 mm^3^
	Orientation	Transverse	Transverse
	Field of view	240 mm × 240 mm	240 mm × 217 mm
	Number of slices	50	50
	Parallel imaging	None	GRAPPA × 3
	TI/TR/refocusing flip angle	89 ms/4800 ms/150°	89 ms/4500 ms/150°
	Turbo factor	17	17
	Scan time	2 min 54 s	1 min 14s
3D FLAIR	Resolution	1 × 1 × 1.1 mm^3^ (interpolated to 0.5 x 0.5 x 1.1 mm3)	1.1 × 1.1 × 1.1 mm^3^
	Orientation	Sagittal	Sagittal
	Field of view	256 mm × 248 mm	280 mm × 254 mm
	Number of slices	176	176
	Parallel imaging	GRAPPA × 2	Wave‐CAIPI 3x2
	TI/TE/TR	1800 ms /393 ms/5000 ms	1800 ms/393 ms/5000 ms
	Turbo factor	265	242
	Scan time	5 min 32 s	1 min 59 s
3D SWI	Resolution	0.9 × 0.9 × 2.3 mm^3^	0.8 × 0.8 × 2.1 mm^3^
	Orientation	Transverse	Transverse
	Field of view	230 mm × 208 mm	280 mm × 210 mm
	Number of slices	64	72
	Parallel imaging	GRAPPA × 2	Wave‐CAIPI 3x2
	TE/TR/flip angle	20 ms/27 ms/15°	21 ms/30 ms/15°
	Scan time	4 min 1 s	1 min 39 s

Abbreviations: 2D, two‐dimensional; 3D, three‐dimensional; FLAIR, fluid‐attenuated inversion recovery; GRAPPA, GeneRalized Autocalibrating Partially Parallel Acquisition; SWI, susceptibility weighted imaging; TE, echo time; TI, inversion time; TR, repetition time; wave‐CAIPI, wave controlled aliasing in parallel imaging.

^*^Twenty individuals underwent fast T1w imaging without fat‐saturation.

### 2.3 Image evaluation

Image evaluation was conducted by three neuroradiologists with 35, 2.5, and 2 years of experience as a consultant. The de‐identified image datasets of 92 individuals were uploaded to a web‐based platform^30^ designed to enable blinded evaluation and were structured across four sessions. In each session, clinical and fast scans of the same individual were presented separately and in random order, with at least 15 scans from other participants interspersed to minimize recall bias. In addition, a subset of 20 randomly chosen clinical scans was shown again to each neuroradiologist (across sessions), again with at least 15 scans from other participants in between scans of the same individual. Raters were blinded to scan protocol, sequence parameters, and patient information—except for age and the fact that the scan was acquired as part of routine cognitive clinic investigations.

The imaging interface allowed for manual adjustments of image scaling widths and levels to obtain optimal contrast, and incorporated a “forced‐choice” questionnaire that did not permit users to go back and revise their ratings for previously submitted cases (see Figure S1 for a screenshot). We aimed to minimize instructions provided to the raters; however, the questionnaire followed a structured approach (see Table S1).^11^, ^31^ Briefly, it included questions about pathology potentially amenable to surgical intervention, vascular disease, and brain volume loss indicative of a neurodegenerative disorder. Reference materials for visual rating scales were supplied for the medial temporal lobe atrophy (MTA) scale^32^, the Koedam^33^ scale for posterior cortical atrophy, and the Fazekas^34^ scale for deep white matter hyperintensities.

**RESEARCH IN CONTEXT**

1. **Systematic review**: We searched PubMed for studies on fast magnetic resonance imaging (MRI) implementations including wave‐controlled aliasing in parallel imaging (wave‐CAIPI) techniques. Single‐sequence comparative studies reported encouraging results for image quality. Two studies found wave‐CAIPI T1‐weighted sequences similar to standard acquisitions using morphometry measures or atrophy visual ratings. However, prior research has been limited by relatively small sample sizes, mixed pathologies, or not assessing a full protocol in clinical settings.
2. **Interpretation**: Dementia MRI protocols that incorporate ultra‐fast three‐dimensional sequences such as those enabled by wave‐CAIPI are comparable to standard‐of‐care clinical scans and are a viable option for improving access to MRI, thereby aiding Alzheimer's disease diagnosis and treatment eligibility.
3. **Future directions**: Fast protocols will need to be optimized for different MRI systems and field strengths and are likely to be improved by deep learning–based reconstruction methods. Reducing time into/out of the scanner would add further value to accelerated scans. This work has implications for other neurological disorders.

Except where there were findings suggestive of non‐AD neurodegenerative disease, the raters were asked to determine whether the scan met exclusion criteria for amyloid‐lowering DMTs, such as five or more microhemorrhages, superficial siderosis, macrohemorrhages, vasogenic edema, severe white matter hyperintensities (Fazekas Grade 3), multiple lacunes, and/or cerebral infarcts involving a major vascular territory.^8^, ^9^ All neuroradiologists had prior experience in the use of the visual rating scales, and no additional training was provided.

Although the primary focus of the study was on the neuroradiologists' assessments, a neurologist with a special interest in cognitive disorders and with 7 years of experience as a consultant provided a supplementary assessment of the scans. By including this additional rater (and Supplementary Analysis), we aimed to explore the reliability of the fast scan in a more heterogenous group of raters, reflecting that neurologists routinely review patient scans. This rater assessed every clinical scan on two separate occasions and reviewed the fast scans once, in one continuous session. Scans from the same individual were separated by at least 15 other cases.

### 2.4 Clinical diagnoses

The clinical diagnosis was taken from the clinical records using all available clinical information and diagnostic guidelines and was independent of the scan assessments.^35^, ^36^, ^37^, ^38^, ^39^, ^40^, ^41^, ^42^, ^43^, ^44^

### 2.5 Aims of the study

Our aim was to determine the reliability of the image assessment between scan types and raters. Reliability was evaluated by measuring the observed level of agreement corrected for chance agreement. We used the Gwet's AC_1_ statistic (and its weighted version AC_2_), which is considered more robust than Cohen's κ to conditions of low category prevalence and/or marginal homogeneity.^45^, ^46^

The primary variable of interest was diagnosis on the scan, operationalized as a nominal variable with three categories: (1) normal, (2) AD, or (3) other diagnoses, which was derived from the questionnaire. The secondary variables were (1) the average of the right and left MTA scores^32^; (2) the Koedam score^33^; (3) the Fazekas score^34^; (4) a microhemorrhage score using a four‐point ordinal scale: no microhemorrhage, 1 to 4, 5–8, and 9 or more microhemorrhages; and (5) radiological eligibility for amyloid‐lowering DMTs.^47^ The latter was recoded as a binary variable (the instances where the rater felt that there was not enough confidence to assess eligibility were recoded as not meeting criteria for DMT). All ordinal scores were analyzed with quadratic weights to place greater penalties on larger disagreements (using the AC_2_ statistic).^48^

To determine reliability across neuroradiologists’ assessments we performed two main sets of analyses. First, we estimated the two inter‐rater reliability coefficients for each scan type (“inter‐rater intra‐fast scan” and “inter‐rater intra‐clinical scan”), as well as the intra‐rater inter‐scan type reliability coefficient (between clinical and fast scans). We estimated the overall inter‐rater reliability between neuroradiologists’ assessments for ratings of the given scan type, followed by pairwise comparisons between each pair of raters. For overall intra‐rater inter‐scan type reliability, we pooled the scores from all raters to minimize the reliance on any single individual's ratings, as described elsewhere,^46^ followed by calculation of reliability for each individual rater. We then compared reliability estimates by calculating (1) the ratio of the inter‐rater intra‐fast scan coefficient to the inter‐rater intra‐clinical scan coefficient (to address the question: is consistency between raters as good for the fast scans as for the clinical scans?), and (2) the ratio of the intra‐rater inter‐scan type coefficient to the inter‐rater intra‐clinical scan coefficient (to address the question: is the consistency of an individual rater assessing fast and clinical scans as good as the consistency of different raters assessing the same clinical scan?).

Second, for the subset of clinical scans that was assessed by the raters on two different occasions, we estimated the intra‐rater intra‐clinical scan reliability between the two assessments of the clinical scans, as well as the two estimates for the intra‐rater inter‐scan type reliability between the fast scan and the first and second assessment of the clinical scan.

To demonstrate the reliability of the fast scan, we sought to confirm that (1) inter‐rater reliability estimates were similar for clinical and fast scans, (2) assessments between the two scan types would show greater reliability than that seen among different raters using the standard‐of‐care clinical scan, and (3) that the reliability within rater comparing the clinical scan on two different occasions would match the within‐rater reliability between clinical and fast scans.

All analyses initially used neuroradiologists ratings. An exploratory analysis was then conducted that included the ratings from the neurologist alongside those of the neuroradiologists.

### 2.6 Statistical analysis

The statistical analysis was performed in R, version 4.4.2.^49^ Continuous variables with a normal distribution are summarized by mean and SD, whereas skewed continuous variables are reported as median and interquartile range (IQR). Categorical variables are presented as total numbers and percentages.

Percent agreement and the chance‐corrected AC_1_ and AC_2_ coefficients were estimated using the “irrCAC” package, version 1.0.0.^50^ Bias‐corrected and accelerated (BCa) 95% bootstrap confidence intervals (CIs) for these coefficients and for the ratios between coefficients were generated with 10,000 bootstrap samples at the subject level, using the “boot” package, version 1.3‐31.^51^, ^52^ The bootstrap CIs for the ratios were calculated on the log ratio scale, as this would be expected to be more normally distributed, and exponentiated for interpretation. Plots were generated using “ggplot2”, version 3.5.1.^53^, [Table alz70341-tbl-0002]

The original power calculation was based on the ability to show that the assessment of the fast scan had excellent reliability with assessments using standard‐of‐care clinical scans. We estimated that 130 participants would be needed to provide 80% power to demonstrate that κ was above the[Fig alz70341-fig-0001] threshold of 0.8 for minimal acceptable agreement between assessments with a one‐sided 95% CI, assuming that the true κ was 0.9. Subsequently, based on pilot data, we estimated that to compare intra‐rater reliability between scan types with inter‐rater reliability on the clinical scans, a sample size of at least 50 participants would be sufficient to have 90% power to demonstrate superiority of the intra‐rater inter‐scan versus inter‐rater intra‐clinical coefficients.

## 3 RESULTS

### 3.1 Participants

**FIGURE 1 alz70341-fig-0001:**
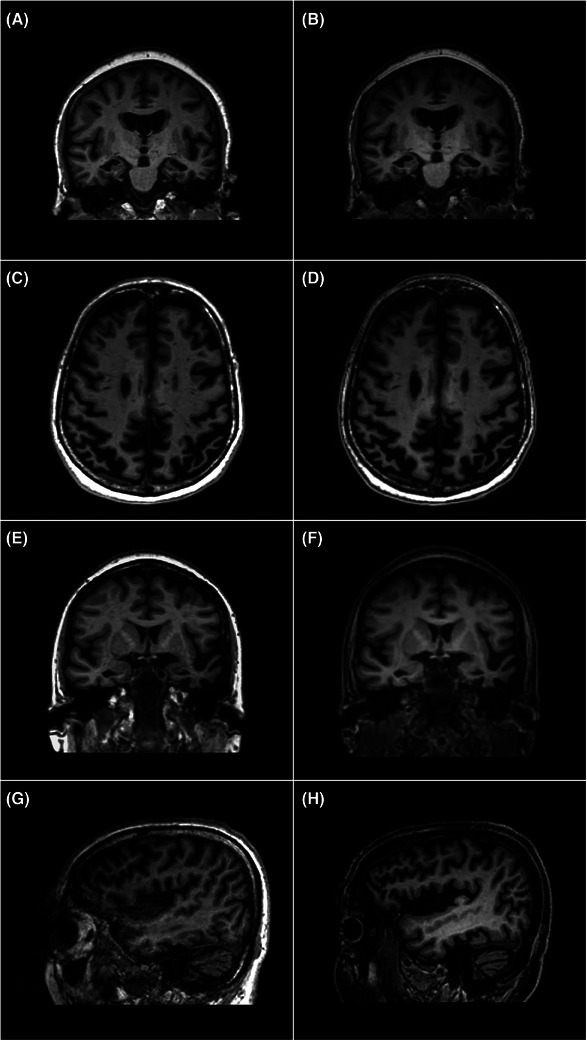
Examples of MRI T1‐weighted sequences from the clinical (A, C, E, G) and fast (B, D, F, H) protocols. A and B present coronal views from a 60‐year‐old female participant with bilateral hippocampal atrophy. C and D show axial views from a 59‐year‐old male participant, illustrating severe atrophy in the parietal lobes. Both participants underwent confirmatory CSF testing for AD. E and F depict coronal views from a 62‐year‐old female participant demonstrating marked asymmetrical left‐sided anterior temporal volume loss, consistent with semantic variant primary progressive aphasia. G and H illustrate sagittal views from a 70‐year‐old male participant with *C9orf72*‐related frontotemporal dementia, showing generalized volume loss, which is particularly prominent in the frontal and temporal lobes. The topographic distribution of brain volume loss is similar in the corresponding clinical and fast scans. AD, Alzheimer's disease; CSF, cerebrospinal fluid; MRI, magnetic resonance imaging.

**FIGURE 2:**
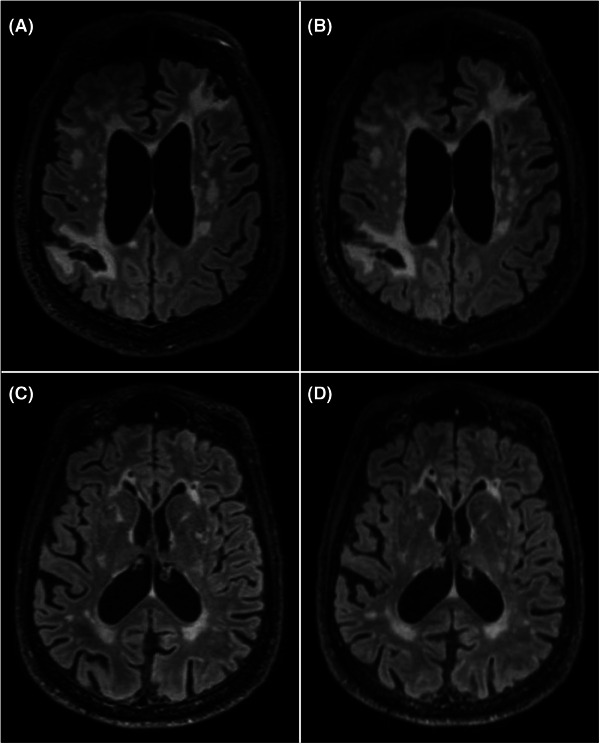
Examples of MRI FLAIR sequences from the clinical (A, C) and fast (B, D) protocols. A and B correspond to axial views from a 64‐year‐old male showing generalized brain volume loss, two areas in the left frontal and right parietooccipital lobes consistent with mature infarcts and a moderate degree of small vessel disease. C and D illustrate axial views of a 74‐year‐old male with partially confluent white matter signal abnormality and bilateral subcortical inferior frontal mature infarcts in keeping with small vessel disease. The imaging features of vascular disease burden appears similarly conspicuous on the clinical (A and C) and fast (B and D) FLAIR sequences. FLAIR, fluid‐attenuated inversion recovery; MRI, magnetic resonance imaging.

**FIGURE 3:**
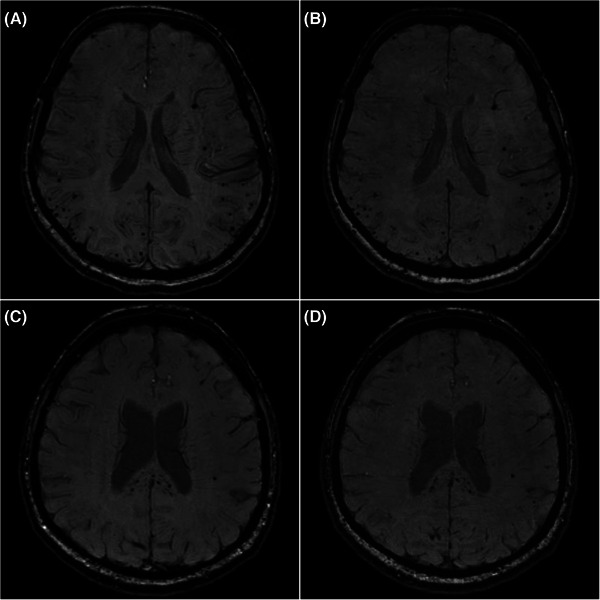
Examples of MRI SWI sequences from the clinical (A and C) and fast (B and D) protocols. A and B depict axial views from a 60‐year‐old female participant with many lobar microhemorrhages, in keeping with cerebral amyloid angiopathy. C and D illustrate axial views from a 52‐year‐old male participant with critical illness‐associated microhemorrhages with conspicuous involvement of the corpus callosum following severe COVID‐19. COVID‐19, coronavirus disease 2019; MRI, magnetic resonance imaging; SWI, susceptibility‐weighted imaging.

Two of the 92 participants were excluded from the analysis due to incorrect image acquisition (see Figure S2). The median age of the remaining 90 individuals was 62 (IQR 57–67); 41 (46%) were female (see Table 2 and Table S2). For four participants, one or more sequences from the clinical scan were repeated due to severe motion artifacts, whereas none of the fast sequences required repetition. In four other cases, the fast protocol was acquired in its entirety after the clinical protocol (i.e., not interleaved), as technical difficulties prevented the prespecified order of sequence acquisition. Figures 1, 2, 3 show examples of clinical and fast scans.

**TABLE 2 alz70341-tbl-0002:** Participant characteristics and clinical diagnoses.

Characteristic	Value, *n* = 90
Age, years*	62, IQR 57–67
Female	41 (45.6)
Clinical diagnoses	
No evidence for neurodegenerative disease, structural abnormality, or vascular burden	37 (41.1)
AD	19 (21.1)
Frontotemporal lobe degeneration	11 (12.2)
Behavioral variant frontotemporal dementia	4 (4.4)
Semantic variant primary progressive aphasia	3 (3.3)
Right temporal lobe variant of frontotemporal lobe degeneration	2 (2.2)
Progressive non‐fluent aphasia	2 (2.2)
Vascular cognitive impairment or mixed disease	8 (8.9)
Dementia with Lewy bodies	4 (4.4)
Cerebral amyloid angiopathy (without evidence of co‐pathology of AD on the scan)	2 (2.2)
Other diagnoses—see Table S2.	9 (10)

*Note*: Unless otherwise specified, data are numbers of participants, with percentages in parentheses.

Abbreviations: AD, Alzheimer's disease; IQR, interquartile range.

* Age is reported as the median, IQR.

### 3.2 Reliability analyses

Inter‐rater reliability among neuroradiologists was similar for the clinical and fast scans (see Table 3). For diagnosis, the *κ̂*_G1_ coefficients were 0.54 (95% CI: 0.45–0.62) for the fast scan and 0.58 (95% CI: 0.50–0.66) for the clinical scan, giving a ratio between the two estimates of 0.92 (95% CI: 0.82–1.04). For the scores on MTA, Koedam, and Fazekas scales, and estimations of microhemorrhages, the inter‐rater reliability on the fast protocol was comparable to that of the clinical protocol, with all *κ̂*_G2_ coefficient ratios being close to one. Likewise, for radiological eligibility for DMTs, the *κ̂*_G1_ coefficients for the fast and clinical scans were very similar, with a ratio of 1.02 (95% CI: 0.97–1.06).

**TABLE 3 alz70341-tbl-0003:** Reliability between neuroradiologists on the clinical scan, on the fast scan, and between scan type across raters.

Question:	How reliable is the assessment among raters on the clinical scan?		How reliable is the assessment among raters on the fast scan?			How reliable is the assessment between clinical and fast scans?		
	Inter‐rater intra‐clinical scan reliability		Inter‐rater intra‐fast scan reliability			Pooled intra‐rater inter‐scan type reliability		
	*p_a_*	*κ̂*_G_ (95% CI)	*p_a_*	*κ̂*_G_ (95% CI)		*p_a_*	*κ̂*_G_ (95% CI)	
Diagnosis on the scan	0.72	0.58 (0.50, 0.66)	0.69	0.54 (0.45, 0.62)	0.92 (0.82, 1.04)	0.86	0.80 (0.74, 0.85)	1.37 (1.21, 1.58)
MTA score	0.97	0.90 (0.88, 0.92)	0.97	0.88 (0.85, 0.90)	0.97 (0.95, 0.99)	0.99	0.98 (0.97, 0.98)	1.08 (1.06, 1.10)
Koedam score	0.93	0.79 (0.75, 0.83)	0.93	0.80 (0.75, 0.84)	1.01 (0.97, 1.05)	0.98	0.94 (0.93, 0.96)	1.19 (1.14, 1.25)
Fazekas score	0.96	0.87 (0.85, 0.89)	0.96	0.86 (0.84, 0.88)	0.99 (0.97, 1.01)	0.98	0.95 (0.94, 0.96)	1.09 (1.07, 1.12)
Microhemorrhages	0.98	0.97 (0.96, 0.98)	0.98	0.96 (0.95, 0.98)	0.99 (0.98, 1.00)	0.99	0.99 (0.99, 0.99)	1.02 (1.01, 1.03)
Radiological eligibility for amyloid‐lowering DMTs	0.92	0.88 (0.80, 0.95)	0.93	0.90 (0.83, 0.95)	1.02 (0.97, 1.06)	0.98	0.97 (0.95, 0.99)	1.10 (1.03, 1.19)

*Note*: The first row indicates the question, and the second row is the statistical metric used to address that question. Data correspond to percent agreement (*p_a_*) and *κ̂*_G_ coefficient with 95% CI in parenthesis. *κ̂*_G1_ is used for diagnosis on the scan and radiological eligibility for DMTs in AD; *κ̂*_G2_ is used for visual rating scales and estimations of microhemorrhages.

Abbreviations: AD, Alzheimer's disease; CI, confidence interval; DMTs, disease‐modifying therapies; MTA, medial temporal lobe atrophy.

Ratio 1: Ratio of inter‐rater intra‐fast *κ̂*_G_ to inter‐rater intra‐clinical scan *κ̂*_G_ (95% CI).

Ratio 2: Ratio of intra‐rater inter‐scan type *κ̂*_G_ to the inter‐rater intra‐clinical scan *κ̂*_G_ (95% CI)

Reliability within raters between the fast and clinical scans was consistently higher than reliability between raters, as shown by the higher *κ̂*_G1_ and *κ̂*_G2_ coefficients on the intra‐rater measures (see Table 3). The ratios comparing pooled intra‐rater reliability between scan types with inter‐rater reliability on the clinical scan, along with their 95% CIs, were all above 1, indicating that there was higher reliability when a neuroradiologist assessed clinical and fast scans on two different occasions compared to when the standard‐of‐care clinical scan was reviewed by their colleagues (see Figure S3 for a detailed pairwise analysis and Table S3 for a similar exploratory analysis incorporating ratings from the neurologist).

**FIGURE 4 alz70341-fig-0004:**
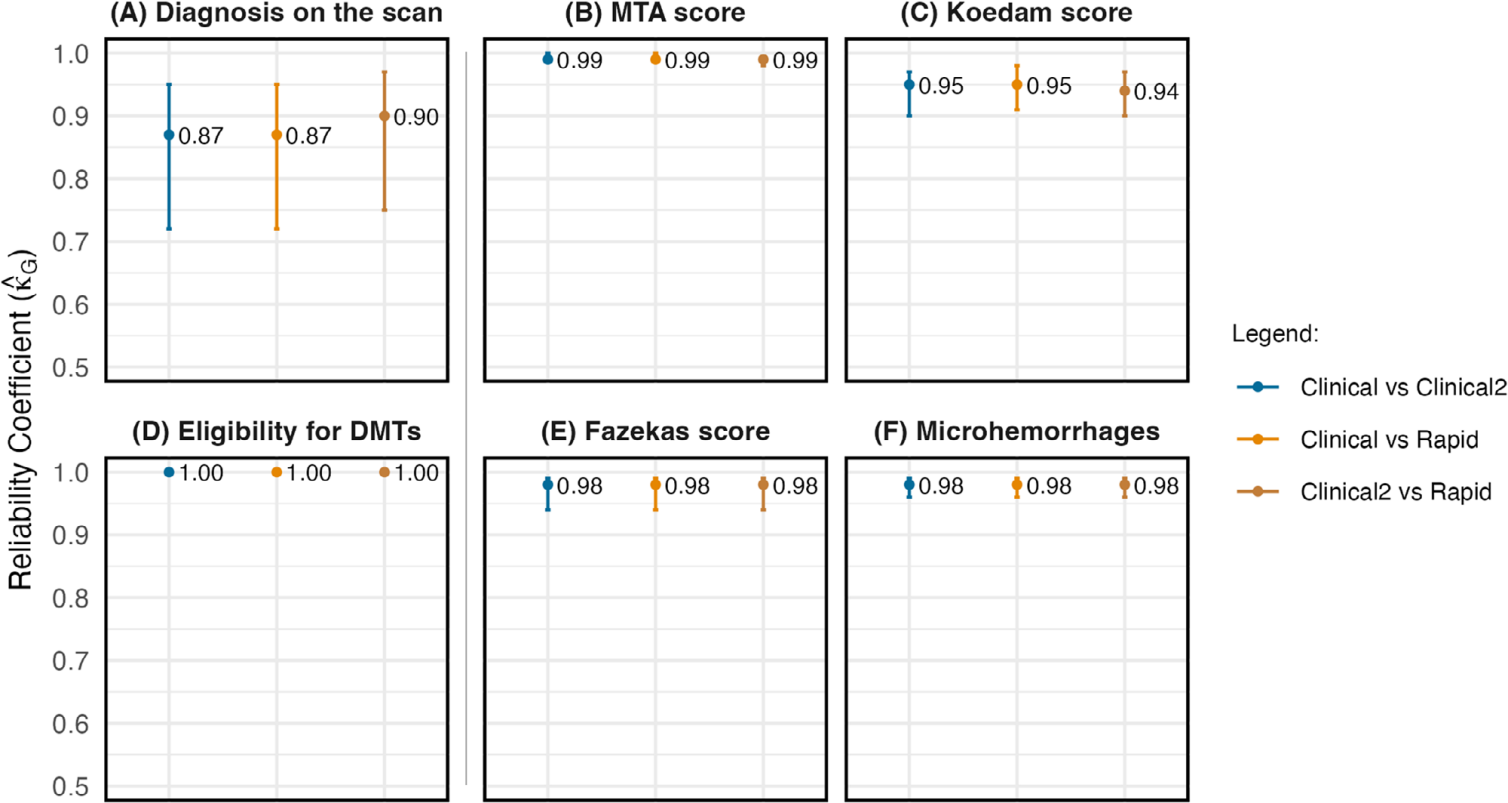
Pooled intra‐rater agreement estimates for the subset of individuals whose clinical scans were evaluated on two separate occasions. Vertical bars represent *κ̂*_G_ coefficients with 95% CIs. A shows *κ̂*_G1_ for diagnosis on the scan (three categories: normal, AD, other diagnoses). B, C, and E show quadratic‐weighted *κ̂*_G2_ for visual scale ratings. D shows *κ̂*_G1_ for radiological eligibility for DMTs in AD. F shows quadratic‐weighted *κ̂*_G2_ for microhemorrhage estimation. AD, Alzheimer's disease; CIs, confidence intervals; DMTs, disease‐modifying therapies.

Figure 4 illustrates the intra‐rater agreement analysis for the subset of individuals whose clinical scans were evaluated on two separate occasions by the neuroradiologists. For each metric, the three *κ̂*_G_ coefficients and their 95% CIs largely overlap, showing similar reliability estimates whether a neuroradiologist reviewed the same clinical scan on two different occasions or assessed the fast scan for the same individual (see Tables S4–S6 for further details).

## 4 DISCUSSION

In this prospective clinical study, the reliability of the neuroradiologists’ assessments between highly accelerated and standard‐of‐care MRI protocols was non‐inferior to the reliability on the standard‐of‐care scan in a real‐world outpatient cognitive service. Both protocols included four core sequences (T1w, T2w, FLAIR, and SWI), with the fast scan reducing total acquisition time by 63% (from 17:39 to 6:29). Each of the four fast sequences took less than 2 min to acquire.

Strikingly, the reliability of the image assessment between clinical and fast scans within raters was consistently higher than the benchmark of reliability between neuroradiologists for the standard‐of‐care clinical scan. Furthermore, the intra‐rater reliability was very similar whether the rating was made on the same clinical scan on two different occasions, or the two ratings were on a fast and a clinical scan from the same patient.

Taken together, these results mean that whether an individual had a fast scan instead of the standard‐of‐care clinical scan introduced less variability than the scan being assessed by different neuroradiologists. Even for the same neuroradiologist, the fast versus clinical variability was no greater than the same clinical scan viewed on two separate occasions. Our results are consistent with previous studies evaluating wave‐CAIPI that investigated single sequences for image quality^23^, ^25^, ^54^ or visual rating scales and morphometric measures,^24^, ^55^ but it is important to note that we evaluate the performance of a more comprehensive fast diagnostic protocol, such as seen typically in the diagnostic setting.

One of the main strengths of our study was its real‐world approach to patient recruitment. We enrolled consecutive individuals attending the outpatient service in whom an MRI brain scan was planned as part of their routine clinical assessment—that is, an unselected group and no distortion of clinical practice. Similarly, the assessment of scans was designed to reflect typical workflows, where decisions are made by a single radiologist rather than through consensus. By examining a heterogeneous, clinically typical patient population under conditions that mirror clinical practice, our study maximizes both the generalizability of the findings and their potential for translation into clinical practice.^56^, ^57^ We anticipate that our findings would generalize to other centers using similar scanner hardware and protocol specifications, specifically 3T systems with comparable head coil configuration; it is likely that less time saving is possible at lower field strengths (e.g., 1.5T).

We also assessed whether accelerating the scans was detrimental to the assessment of eligibility for amyloid‐lowering DMTs. There was no difference between the reliability of assessments based on the fast and the clinical protocols, and, here too, the variability between scans was significantly less than between raters on the clinical scan. This is important given that a potential bottleneck to accessing DMTs is the availability of MRI. It may also be that highly accelerated scans would be just as effective at the detection of amyloid‐related imaging abnormalities (ARIA), which would facilitate safe implementation of these new therapies. It is important to note, however, that SWI acquisitions (whether accelerated via wave‐CAIPI or not) are generally more sensitive to microhemorrhage detection than T2*‐weighted gradient recalled‐echo sequences used in clinical trials. Further research is needed to understand how incorporating SWI into accelerated protocols affects patient eligibility and ARIA monitoring.

Our initial sample size was adjusted following optimization of the fast protocol acquisition parameters, as the optimized sequences showed very high correspondence to the clinical sequences on side‐by‐side visual inspection. Based on this, we selected inter‐rater reliability on the clinical scans as a benchmark for comparison, which allowed a reduction in the required sample size.

Our study is not without limitations. First, participants were recruited from a specialist cognitive clinic, and scans were acquired using a single imaging system (Siemens MAGNETOM Prisma Fit 3T). As a result, our cohort is likely to be younger (although individuals younger than 50 years of age were excluded), to include less common clinical conditions, and to have less cerebrovascular disease compared to the typical local memory service population. We also acknowledge that, as the study was conducted at a single, specialist center, there may be limited racial, ethnic, and socioeconomic representation. A limitation is that such data were not collected. Furthermore, scans were assessed by neuroradiologists rather than general radiologists. Nevertheless, it is plausible that older populations would particularly benefit from faster acquisitions, as comorbidities may make it more difficult for them to remain still in the scanner. In addition, neuroradiologists, as neuroimaging experts, are more likely to be discerning of brain scan differences than general radiologists. Our exploratory analysis, which included a non‐imaging specialist, did not suggest that scan appearance differences are likely to be a substantial factor affecting the diagnostic utility of the fast scans. For practical reasons, we chose to assess a single method (wave‐CAIPI) for accelerating MRI. However, we believe our findings are likely to be generalizable; other methods, such as compressed sensing and artificial intelligence–enabled reconstruction, should offer similar or potentially greater acceleration benefits in this rapidly advancing field. Further research is needed to evaluate fast imaging techniques across different vendors and field strengths. Inevitably different scanner hardware and software performance will influence the degree of acceleration that allows diagnostically useful acquisitions. Although it seems likely that meaningful acceleration will be possible across most contemporary MRI scanners, this needs full assessment in the context of a multi‐center evaluation.[Table alz70341-tbl-0003], [Fig alz70341-fig-0004]

Second, we did not formally assess scans for image quality or artifacts, as the primary aim of this study was to evaluate the diagnostic reliability of the fast scan. However, any substantial image quality degradation would likely have been reflected in reduced reliability estimates. In addition, and as per routine clinical practice, radiographers were instructed to immediately assess sequence quality and re‐acquire scans if necessary. Again, to minimize disruption to clinical workflows, the acquisition order was kept consistent throughout the study, with the fast sequence acquired prior to the corresponding clinical standard sequence, which may also be a limitation of our study. With these caveats, we note that in four cases, one or more clinical sequences required re‐acquisition due to motion artifacts, whereas no fast sequences needed repetition.

Third, we did not assess overall MRI slot duration, which includes time allocated to getting into and out of the scanner as well as localizing (scout) sequences. These essential steps become a more substantial proportion of the overall patient visit as the core scan protocol duration is reduced, and optimization of these steps is therefore increasingly worthy of focus.

Finally, it is worth noting that we chose only to include four, admittedly key, sequences in the scan protocols.^31^ There may be instances when individuals will need additional sequences, for example, contrast‐enhanced or diffusion‐weighted imaging. Nevertheless, we believe that the sequences chosen are the workhorse sequences for the clinical context. Although some patients may need to return for a further scan with additional sequences, we believe that our approach is a pragmatic one to address the huge demand for MRI.

Our study serves as a proof of concept that accelerated sequences within an optimized protocol can achieve substantial time savings without compromising diagnostic utility. Reducing the time needed for scans could increase access to MRI and reduce costs. Shorter scans should improve patient experience, many of whom have difficulty remaining still for long scans. Highly accelerated MRI protocols could help stretched clinical services to deliver a timely diagnosis to the increasing numbers of individuals with cognitive concerns and to assess eligibility for DMTs for AD before individuals are too severely affected to benefit.

## CONFLICT OF INTEREST STATEMENT

C.J.M. has received consulting fees from Biogen, Roche, Lilly, Eisai, Novartis, Neurimmune, Merck Sharp & Dohme (MSD), and GlaxoSmithKline (GSK), and is on the advisory board for Immunobrain. C.S.R.B. has no conflicts of interest. D.C.A. is a shareholder of Queen Square Analytics Limited, and has received grants from UK Research and Innovation (UKRI), the Wellcome Trust, and the National Institute for Health and Care Research University Collehe London Hospitals Biomedical Research Centre (NIHR UCLH BRC). D.L.T. has received support from the NIHR UCLH BRC and Alzheimer's Society. D.M., and E.A.L. have no conflicts of interest. F.B. is a Steering Committee or Data Safety Monitoring Board member for Biogen, Merck, Eisai, and Prothena, and is an advisory board member for Combinostics, Scottish Brain Sciences, and Alzheimer Europe. F.B. is a consultant for Roche, Celltrion, Rewind Therapeutics, Merck, and Bracco, and has research agreements with ADDI, Merck, Biogen, GE Healthcare, and Roche. F.B. is co‐founder and shareholder of Queen Square Analytics Limited. F.B. is supported by the NIHR UCLH BRC. F.U. has no conflicts of interest. G.J.M.P. receives salary from, is a director of, and holds stock in Bioxydyn Limited. G.J.M.P. is a director of and holds stock in Quantitative Imaging Limited. G.J.M.P. is a director of and holds stock in Queen Square Analytics Limited. G.J.M.P. has received research grant funding from Biogen, GSK, Eli Lilly, and Siemens. H.R.C. has no conflicts of interest. H.R.J. has been supported by Rosetrees Trust, has received royalties from Springer Nature Publishers and has participated on data safety monitoring/advisory board for Merck. J.M.N. is supported by NIHR UCLH BRC. M.B., and M.R.G. have no conflicts of interest. N.C.F. has received consulting fees from Eisai, F. Hoffmann‐La Roche, Eli Lilly, and Biogen, and is an advisory board member for Biogen and Abbvie. N.M. is supported by NIHR UCLH BRC. O.N. has no conflicts of interest. Author disclosures are available in the Supporting Information.

## CONSENT STATEMENT

All human subjects involved in this study provided written informed consent.

## Supporting information



Supporting Information

Supporting Information
