# Supplementary material for: Ultra‐fast MRI for dementia diagnosis and treatment eligibility: A prospective study
Source: Alzheimers Dement. 2025 Jun 11;21(6):e70341. doi: 10.1002/alz.70341 (PMC12159316; doi:10.1002/alz.70341)
Supplement: Supplementary file 1 — Supporting Information [file ALZ-21-e70341-s002.docx]

| Supplementary Table 1. Questionnaire |
| --- |
| Q1. Please exclude “surgical” pathology as a potential cause for cognitive decline. |
| 0: There is no evidence for “surgical pathology” as a potential cause for cognitive impairment. (Go to Q3)  1: “Surgical pathology” may be a contributing cause for cognitive decline. (Go to Q2) |
| Q2. Please choose one or more of the following: |
| 0: Lesion with mass effect (Go to Q3)  1: Normal pressure hydrocephalus (Go to Q3)  2: Subdural haematoma (Go to Q3)  3: Other: (Go to Q3) |
| Q3. Please provide a Fazekas scale score for deep white matter hyperintensities. (Examples of scorings using the Fazekas’ rating scale available for reference.) |
| 0: Fazekas 0 – no lesions or a single punctuate WMH lesion (Go to Q4)  1: Fazekas 1: multiple punctate lesions (Go to Q4)  2: Fazekas 2: beginning confluency of lesions (bridging) (Go to Q4)  3: Fazekas 3: large confluent lesions (Go to Q4) |
| Q4. Are there any large-vessel infarcts, lacunes or macrohaemorrhages? |
| 1: Yes (Go to Q5)  2: No (Go to Q6) |
| Q5. Please provide a count and description for the vascular lesions mentioned in the previous question. |
| Large-vessel infarcts: [count and description]  Lacunes: [count and description]  Macrohaemorrhages: [count and description] |
| Q6. Please provide an estimate for the number of cerebral microbleeds. |
| 0: None (Go to Q7)  1: 1-4 (Go to Q7)  2: 5-8 (Go to Q7)  3: 9 or more (Go to Q7) |
| Q7. Please assess the scan for probable vascular cognitive impairment as per NINDS-AIREN criteria and choose one of the following:  (The NINDS-AIREN criteria are available for reference.) |
| 0: Radiological findings do not meet topography / severity criteria (Go to Q8)  1: Radiological findings meet criteria for probable vascular cognitive impairment due to small-vessel disease including bilateral thalamic lesions (Go to Q8)  2: Radiological findings meet criteria for probable vascular cognitive impairment due to large-vessel disease (Go to Q8)  3: There is fulfilment of radiological criteria for both small-vessel and large-vessel disease (Go to Q8) |
| Q8. Is there evidence for probable cerebral amyloid angiopathy as per Boston 2.0 criteria?  (The Boston 2.0 criteria are available for reference.) |
| 0: No (Go to Q9)  1: Yes (Go to Q9) |
| Q9. Please provide an MTA score for the right hippocampus.  (MTA criteria are available for reference with anatomical landmark examples.) |
| 0: MTA 0 – no atrophy (Go to Q10)  1: MTA 1 – only widening of choroid fissure (Go to Q10)  2: MTA 2 – also widening of temporal horn of lateral ventricle (Go to Q10)  3: MTA 3 – moderate loss of hippocampal volume (decrease in height) (Go to Q10)  4: MTA 4 – severe volume loss of hippocampus (Go to Q10) |
| Q10. Please provide an MTA score for the left hippocampus. |
| 0: MTA 0 – no atrophy (Go to Q11)  1: MTA 1 – only widening of choroid fissure (Go to Q11)  2: MTA 2 – also widening of temporal horn of lateral ventricle (Go to Q11)  3: MTA 3 – moderate loss of hippocampal volume (decrease in height) (Go to Q11)  4: MTA 4 – severe volume loss of hippocampus (Go to Q11) |
| Q11. Please provide a single Koedam score for parietal atrophy.  (Koedam’s criteria are available for consulting with anatomical landmark examples.) |
| 0: Koedam 0 – closed sulci of parietal lobes and precuneus. (Go to Q12)  1: Koedam 1 – mild widening of posterior cingulate and parieto-occipital sulci (Go to Q12)  2: Koedam 2 – substantial widening of sulci (Go to Q12)  3: Koedam 3 – extreme widening of posterior cingulate and parieto-occipital sulci (Go to Q12) |
| Q12. Please assess the scan for neurodegenerative disease and choose one of the following: |
| 0: Parenchymal volumes are in keeping with age (Go to Q15)  1: Findings suggestive of Alzheimer’s disease (classical and variants) (Go to Q13)  2: Findings suggestive of frontotemporal lobe degeneration including cases of progressive supranuclear palsy (Go to Q14)  3: Other: [description] (Go to Q15) |
| Q13. Which of these variants is most likely? |
| 0: Hippocampal-predominant / typical Alzheimer’s disease (Go to Q15)  1: Posterior cortical atrophy (Go to Q15)  2: Logopenic progressive aphasia (Go to Q15) |
| Q14. Which of the following is most likely? |
| 0: Behavioural variant FTD (Go to Q16)  1: Progressive non-fluent aphasia (Go to Q16)  2: Semantic variant primary progressive aphasia (Go to Q16)  3: Right-temporal lobe variant of FTLD (Go to Q16)  4: PSP (Go to Q16)  5: Other: [description] (Go to Q16) |
| Q15. From a safety standpoint are there any MRI-based exclusionary findings for anti-amyloid monoclonal antibody therapies?  Pivotal trials have excluded patients with any CNS macrohaemorrhage >10 mm in diameter, more than 4 microhaemorrhages (<10 mm in diameter), evidence of superficial siderosis, evidence of brain vasogenic oedema, significant/severe white matter hyperintensities, multiple lacunar strokes and/or any cerebral infarcts involving a major vascular territory. |
| 0: No (Go to Q15)  1: Yes (Go to Q15)  2: Could not be assessed with confidence (Go to Q15) |
| Q16. Please provide any final comments as required such as incidental findings, artefact, suggestions. |
|  |

| Supplementary Table 2. Participant characteristics and clinical diagnoses: other diagnoses | |
| --- | --- |
| Characteristic | Value, *n* = 90 |
| Cognitive difficulties attributed to excessive alcohol consumption | 2 (2.2) |
| Head injury | 2 (2.2) |
| Presumed limbic-predominant age related TDP-43 encephalopathy (with negative AD biomarkers) | 2 (2.2) |
| Cognitive difficulties likely in the context of neurodevelopment delay | 1 (1.1) |
| Normal pressure hydrocephalus | 1 (1.1) |
| Critical illness-associated microhemorrhages | 1 (1.1) |
| Investigations performed as part of routine clinical practice |  |
| Cerebrospinal fluid biomarkers for Alzheimer’s disease | 19 (21.1) |
| Amyloid PET imaging | 3 (3.3) |
| [123I]-FP-CIT SPECT (DaTscan) imaging | 3 (3.3) |
| 18F-FDG brain PET imaging | 1 (1.1) |
| NOTE. Data are numbers of participants, with percentages in parentheses.  Abbreviations: TDP-43, TAR DNA-binding protein 43; AD, Alzheimer’s disease; PET, positron emission tomography; SPECT, single-photon emission computed tomography | |

| Supplementary Table 3. Reliability between all raters on the clinical scan, on the fast scan, and between scan type across raters | | | | | | | | |
| --- | --- | --- | --- | --- | --- | --- | --- | --- |
|  | *How reliable is the assessment between raters on the clinical scan?* | | *How reliable is the assessment between raters on the fast scan?* | |  | *How reliable is the assessment between clinical and fast scans?* | |  |
|  | Inter-rater intra-clinical scan reliability | | Inter-rater intra-fast scan reliability | |  | Pooled intra-rater inter-scan type reliability | |  |
|  | *p_a_* | *κ̂*_G_ (95% CI) | *p_a_* | *κ̂*_G_ (95% CI) | Ratio of inter-rater intra-fast *κ̂*_G_ to inter-rater intra-clinical scan *κ̂*_G_ (95% CI) | *p_a_* | *κ̂*_G_ (95% CI) | Ratio of intra-rater inter-scan type *κ̂*_G_ to the inter-rater intra-clinical scan *κ̂*_G_ (95% CI) |
| Diagnosis on the scan | 0.71 | 0.58 (0.50, 0.65) | 0.71 | 0.57 (0.50, 0.65) | 0.99 (0.90, 1.09) | 0.86 | 0.80 (0.75, 0.85) | 1.38 (1.25, 1.55) |
| MTA score | 0.97 | 0.89 (0.87, 0.91) | 0.97 | 0.88 (0.86, 0.90) | 0.99 (0.97, 1.01) | 0.99 | 0.97 (0.96, 0.98) | 1.09 (1.07, 1.11) |
| Koedam score | 0.93 | 0.81 (0.78, 0.85) | 0.94 | 0.83 (0.79, 0.86) | 1.02 (0.99, 1.05) | 0.98 | 0.93 (0.92, 0.95) | 1.15 (1.11, 1.20) |
| Fazekas score | 0.95 | 0.85 (0.83, 0.87) | 0.95 | 0.84 (0.82, 0.87) | 0.99 (0.96, 1.01) | 0.98 | 0.94 (0.93, 0.95) | 1.11 (1.08, 1.14) |
| Micro-hemorrhages | 0.98 | 0.97 (0.95, 0.98) | 0.98 | 0.97 (0.95, 0.98) | 1.00 (0.99, 1.01) | 0.99 | 0.99 (0.99, 0.99) | 1.02 (1.01, 1.04) |
| Radiological eligibility for amyloid-lowering DMTs | 0.91 | 0.86 (0.78, 0.93) | 0.93 | 0.90 (0.83, 0.96) | 1.04 (0.98, 1.11) | 0.96 | 0.95 (0.92, 0.97) | 1.10 (1.04, 1.18) |
| NOTE. The first row indicates the question, and the second row is the statistical metric used to address that question. Data corresponds to percent agreement (*p_a_*) and *κ̂*_G_ coefficient with 95% CI in parenthesis. *κ̂*_G1_ is used for diagnosis on the scan and radiological eligibility for DMTs in Alzheimer's disease; *κ̂*_G2_ is used for visual rating scales and estimations of microhemorrhages. | | | | | | | | |
| Abbreviations: CI, confidence interval; DMTs, disease modifying therapies; MTA, medial temporal lobe atrophy | | | | | | | | |

| Supplementary Table 4. Intra-rater reliability for the subset of individuals whose clinical scans were assessed on two separate occasions | | | | | | | |
| --- | --- | --- | --- | --- | --- | --- | --- |
|  |  | Intra-rater between the two assessments of clinical scan | | Intra-rater between fast and first assessment of clinical scan | | Intra-rater between fast and second assessment of clinical scan | |
|  | Rater | *p*_a_ | *κ̂*_G_ (95% CI) | *p*_a_ | *κ̂*_G_ (95% CI) | *p*_a_ | *κ̂*_G_ (95% CI) |
| Diagnosis on the scan | A | 0.95 | 0.92 (0.53-1.00) | 0.89 | 0.84 (0.45-0.93) | 0.95 | 0.92 (0.53-1.00) |
|  | B | 0.80 | 0.71 (0.33-0.87) | 0.85 | 0.79 (0.43-0.93) | 0.85 | 0.78 (0.41-0.93) |
|  | C | 1.00 | 1.00 (N/A) | 1.00 | 1.00 (N/A) | 1.00 | 1.00 (N/A) |
|  | D | 0.90 | 0.86 (0.74-0.94) | 0.87 | 0.81 (0.69-0.89) | 0.90 | 0.85 (0.74-0.94) |
| MTA score | A | 1.00 | 0.99 (0.98-1.00) | 1.00 | 0.99 (0.98-1.00) | 1.00 | 0.99 (0.98-0.99) |
|  | B | 1.00 | 0.99 (0.98-1.00) | 1.00 | 0.99 (0.99-1.00) | 1.00 | 0.99 (0.98-0.99) |
|  | C | 1.00 | 1.00 (1.00-1.00) | 1.00 | 1.00 (0.98-1.00) | 1.00 | 1.00 (0.98-1.00) |
|  | D | 1.00 | 0.99 (0.99-1.00) | 1.00 | 0.99 (0.98-0.99) | 1.00 | 0.99 (0.99-1.00) |
| Koedam score | A | 0.98 | 0.94 (0.86-0.98) | 0.96 | 0.91 (0.82-0.96) | 0.96 | 0.91 (0.84-0.96) |
|  | B | 0.98 | 0.96 (0.90-0.99) | 0.99 | 0.97 (0.92-1.00) | 0.98 | 0.96 (0.89-0.99) |
|  | C | 0.98 | 0.95 (0.87-0.99) | 0.99 | 0.98 (0.90-1.00) | 0.99 | 0.97 (0.88-1.00) |
|  | D | 0.98 | 0.96 (0.94-0.98) | 0.97 | 0.92 (0.86-0.94) | 0.97 | 0.93 (0.87-0.95) |
| Fazekas score | A | 1.00 | 1.00 (N/A) | 1.00 | 1.00 (N/A) | 1.00 | 1.00 (N/A) |
|  | B | 0.98 | 0.96 (0.88-0.99) | 0.98 | 0.95 (0.87-0.99) | 0.98 | 0.96 (0.89-0.99) |
|  | C | 0.99 | 0.98 (0.91-1.00) | 1.00 | 1.00 (N/A) | 0.99 | 0.98 (0.91-1.00) |
|  | D | 0.99 | 0.97 (0.94-0.98) | 0.97 | 0.92 (0.89-0.95) | 0.98 | 0.94 (0.91-0.96) |
| Microhemorrhages | A | 0.98 | 0.97 (0.92-0.99) | 0.99 | 0.99 (0.94-1.00) | 0.99 | 0.98 (0.93-1.00) |
|  | B | 0.98 | 0.98 (0.93-0.99) | 0.99 | 0.99 (0.95-1.00) | 0.99 | 0.99 (0.94-1.00) |
|  | C | 0.99 | 0.99 (0.94-1.00) | 0.98 | 0.96 (0.91-0.99) | 0.98 | 0.97 (0.92-0.99) |
|  | D | 1.00 | 0.99 (0.98-1.00) | 1.00 | 0.99 (0.98-1.00) | 1.00 | 1.00 (0.99-1.00) |
| Radiological eligibility for amyloid-lowering DMTs | A | 1.00 | 1.00 (N/A) | 1.00 | 1.00 (N/A) | 1.00 | 1.00 (N/A) |
|  | B | 1.00 | 1.00 (N/A) | 1.00 | 1.00 (N/A) | 1.00 | 1.00 (N/A) |
|  | C | 1.00 | 1.00 (N/A) | 1.00 | 1.00 (N/A) | 1.00 | 1.00 (N/A) |
|  | D | 0.96 | 0.94 (0.83-0.98) | 0.92 | 0.89 (0.75-0.96) | 0.96 | 0.94 (0.82-0.98) |
| NOTE. Data corresponds to percent agreement (*p_a_*) and *κ̂*_G_ coefficient with 95% CI in parenthesis. *κ̂*_G1_ is used for diagnosis on the scan and radiological eligibility for DMTs in Alzheimer's disease; *κ̂*_G2_ is used for visual rating scales and estimations of microhaemorrhages. Raters A, B, C and D reviewed clinical scans of 19, 20, 19 and 90 individuals. Radiological eligibility for DMTs was assessed for 15, 19, 13, and 72 individuals respectively. | | | | | | | |
| Abbreviations: CI, confidence interval; DMTs, disease modifying therapies; MTA, medial temporal lobe atrophy; N/A, not applicable | | | | | | | |

Supplementary Table 5. Pooled intra-rater reliability for the subset of individuals whose clinical scans were assessed on two separate occasions – for neuroradiologists

|  | Intra-rater between the two assessments of clinical scan | | Intra-rater between fast and first assessment of clinical scan | | Intra-rater between fast and second assessment of clinical scan | |
| --- | --- | --- | --- | --- | --- | --- |
|  | *p*_a_ | *κ̂*_G_ (95% CI) | *p*_a_ | *κ̂*_G_ (95% CI) | *p*_a_ | *κ̂*_G_ (95% CI) |
| Diagnosis on the scan | 0.91 | 0.87 (0.72-0.95) | 0.91 | 0.87 (0.72-0.95) | 0.93 | 0.90 (0.75-0.97) |
| MTA score | 1.00 | 0.99 (0.99-1.00) | 1.00 | 0.99 (0.99-1.00) | 1.00 | 0.99 (0.98-0.99) |
| Koedam score | 0.98 | 0.95 (0.90-0.97) | 0.98 | 0.95 (0.91-0.98) | 0.98 | 0.94 (0.90-0.97) |
| Fazekas score | 0.99 | 0.98 (0.94-0.99) | 0.99 | 0.98 (0.94-0.99) | 0.99 | 0.98 (0.94-0.99) |
| Microhemorrhages | 0.99 | 0.98 (0.96-0.99) | 0.99 | 0.98 (0.96-0.99) | 0.99 | 0.98 (0.96-0.99) |
| Radiological eligibility for amyloid-lowering DMTs | 1.00 | 1.00 (N/A) | 1.00 | 1.00 (N/A) | 1.00 | 1.00 (N/A) |
| NOTE. Data corresponds to percent agreement (*p_a_*) and *κ̂*_G_ coefficient with 95% CI in parenthesis. *κ̂*_G1_ is used for diagnosis on the scan and radiological eligibility for DMTs in Alzheimer's disease; *κ̂*_G2_ is used for visual rating scales and estimations of microhemorrhages. | | | | | | |
| Abbreviations: CI, confidence interval; DMTs, disease modifying therapies; MTA, medial temporal lobe atrophy; N/A, not applicable | | | | | | |

Supplementary Table 6. Pooled intra-rater reliability for the subset of individuals whose clinical scans were assessed on two separate occasions – for all raters

|  | Intra-rater between the two assessments of clinical scan | | Intra-rater between fast and first assessment of clinical scan | | Intra-rater between fast and second assessment of clinical scan | |
| --- | --- | --- | --- | --- | --- | --- |
|  | *p*_a_ | *κ̂*_G_ (95% CI) | *p*_a_ | *κ̂*_G_ (95% CI) | *p*_a_ | *κ̂*_G_ (95% CI) |
| Diagnosis on the scan | 0.91 | 0.86 (0.78-0.92) | 0.89 | 0.83 (0.74-0.90) | 0.91 | 0.87 (0.79-0.93) |
| MTA score | 1.00 | 0.99 (0.99-1.00) | 1.00 | 0.99 (0.98-0.99) | 1.00 | 0.99 (0.99-0.99) |
| Koedam score | 0.98 | 0.96 (0.93-0.97) | 0.97 | 0.93 (0.89-0.95) | 0.97 | 0.93 (0.90-0.95) |
| Fazekas score | 0.99 | 0.97 (0.95-0.98) | 0.98 | 0.94 (0.91-0.96) | 0.98 | 0.95 (0.93-0.97) |
| Microhemorrhages | 0.99 | 0.99 (0.98-0.99) | 0.99 | 0.99 (0.98-1.00) | 0.99 | 0.99 (0.98-1.00) |
| Radiological eligibility for amyloid-lowering DMTs | 0.97 | 0.97 (0.90-0.99) | 0.95 | 0.93 (0.85-0.97) | 0.97 | 0.96 (0.90-0.99) |
| NOTE. Data corresponds to percent agreement (*p_a_*) and *κ̂*_G_ coefficient with 95% CI in parenthesis. *κ̂*_G1_ is used for diagnosis on the scan and radiological eligibility for DMTs in Alzheimer's disease; *κ̂*_G2_ is used for visual rating scales and estimations of microhemorrhages. | | | | | | |
| Abbreviations: CI, confidence interval; DMTs, disease modifying therapies; MTA, medial temporal lobe atrophy | | | | | | |

Supplementary Figure 1.


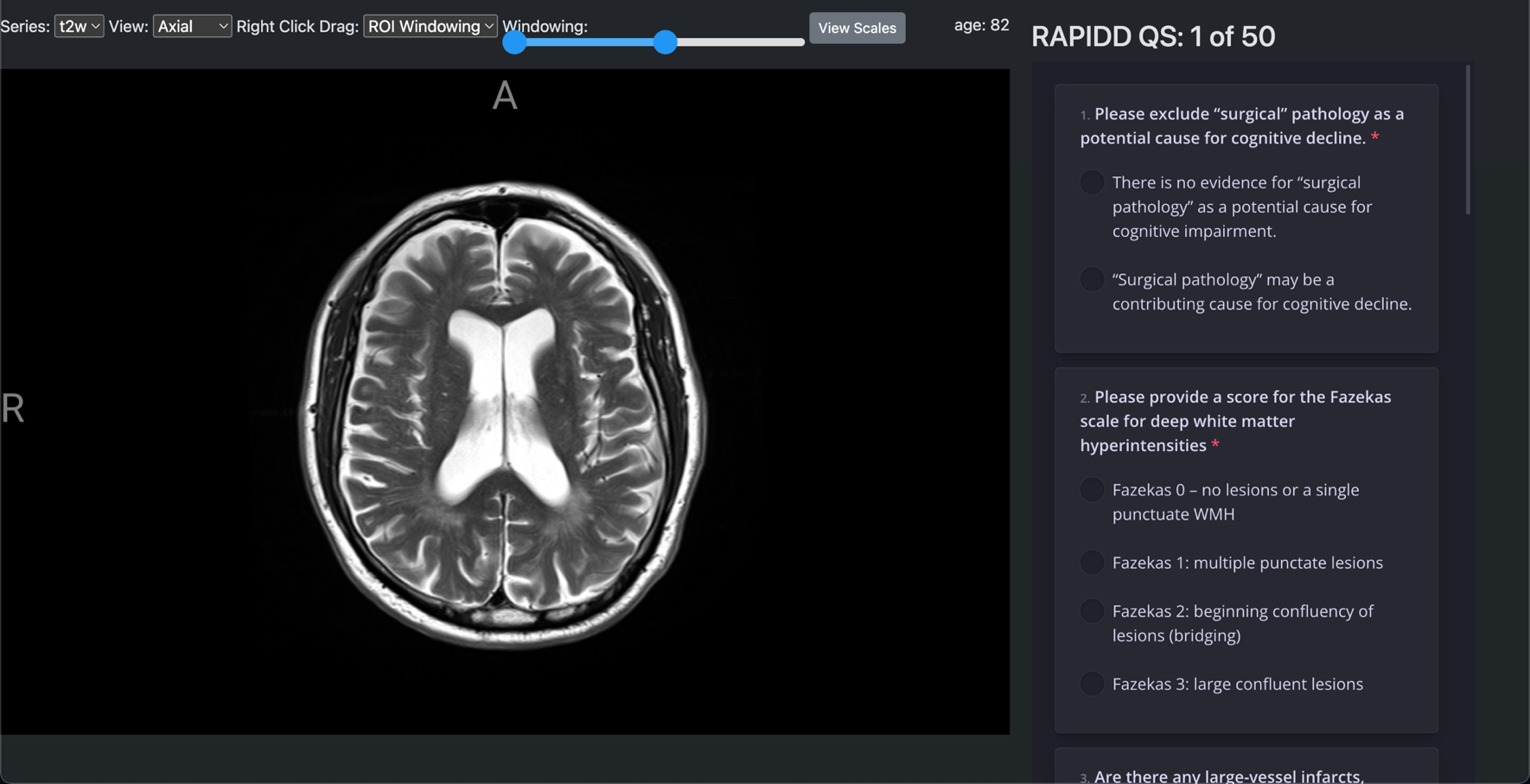


Screenshot of the imaging interface for scan evaluation.

Supplementary Figure 2.


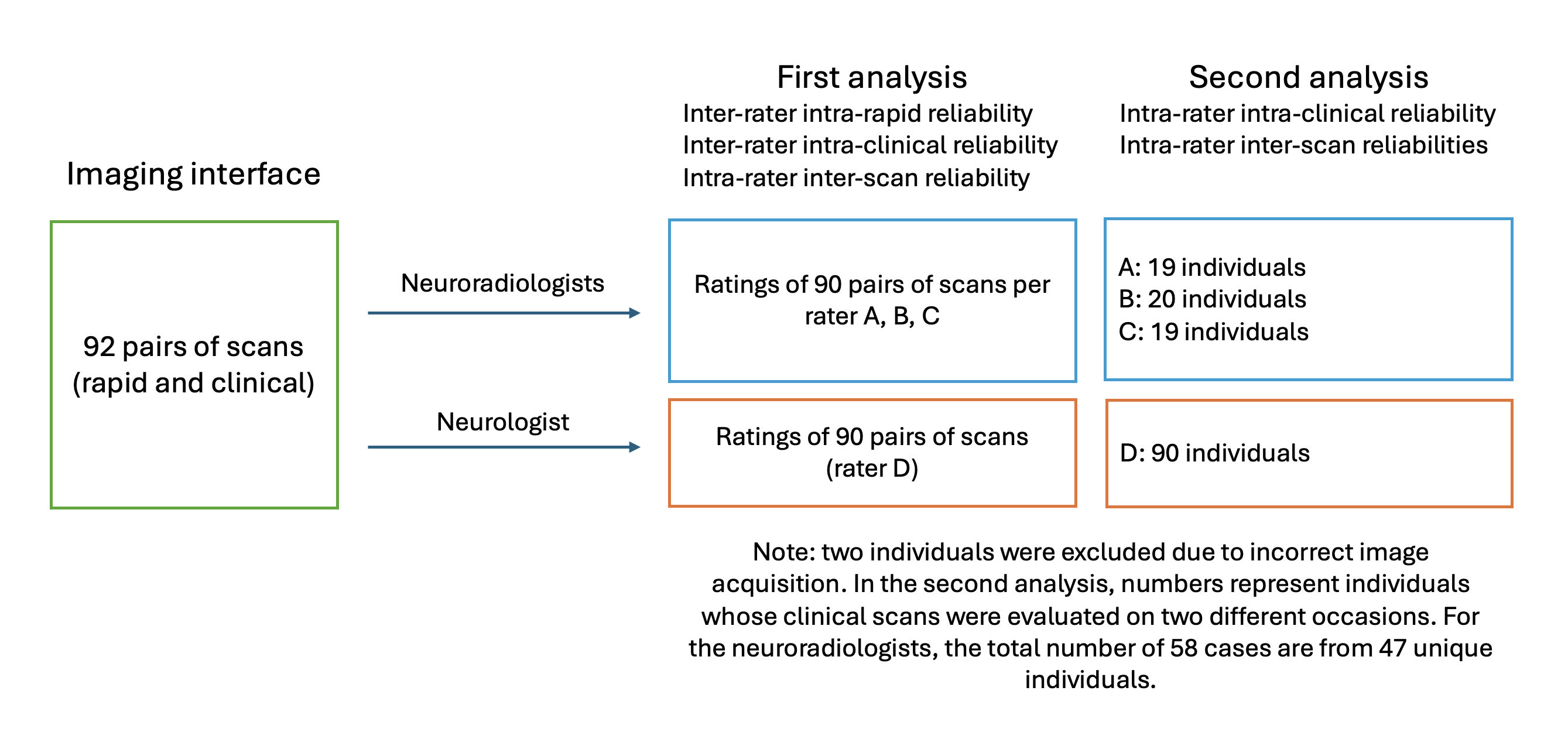


Supplementary Figure 3.


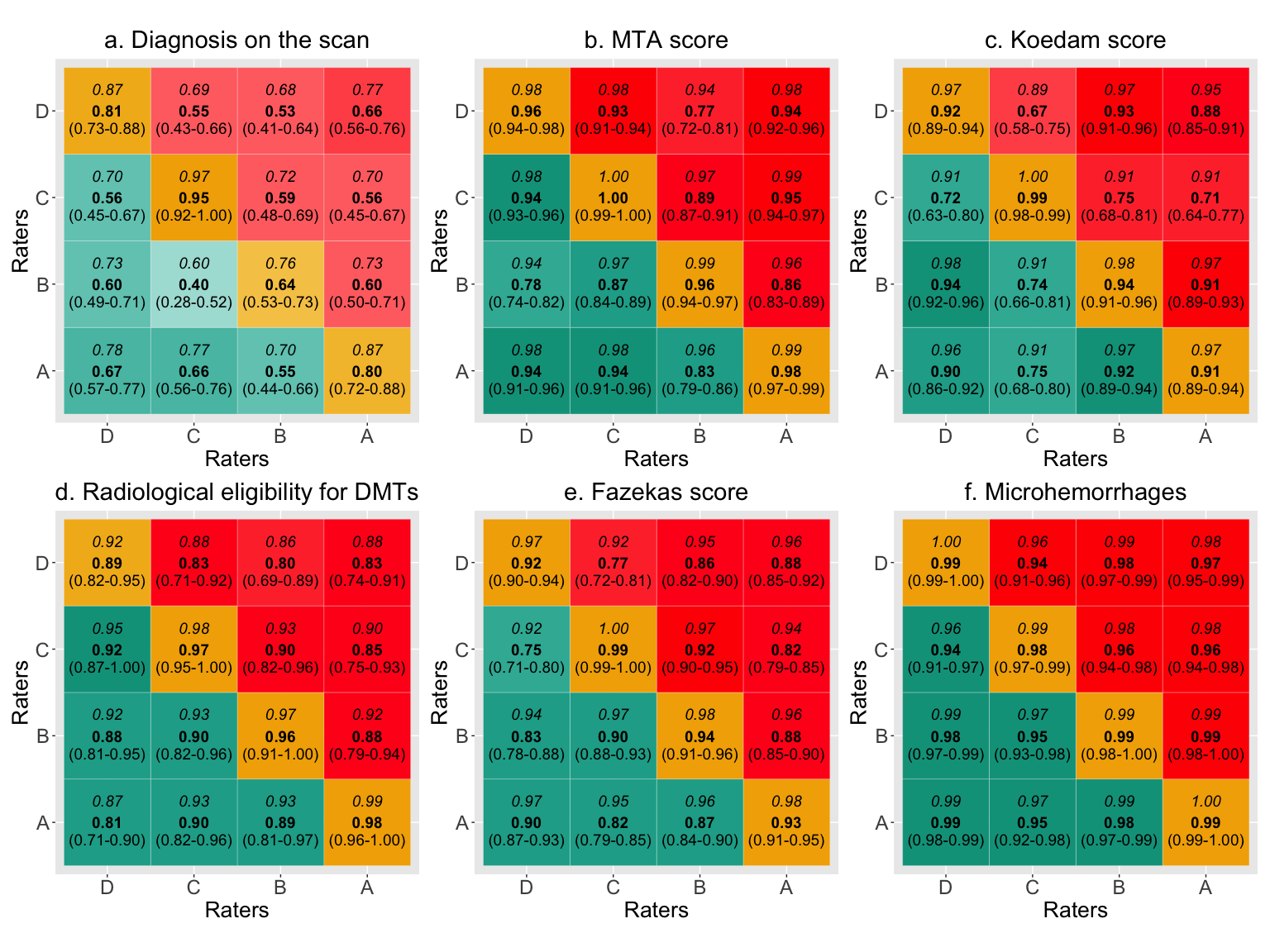


NOTE. Green and red tiles represent pairwise *κ̂*_G_ coefficients between raters for the fast and clinical scan respectively. Yellow tiles represent intra-rater *κ̂*_G_ coefficients between the clinical and fast scan for each rater. Panel a – *κ̂*_G1_ for diagnosis on the scan, panels b, c, e – quadratic-weighted *κ̂*_G2_ for visual scale ratings, panel d – *κ̂*_G1_ for radiological eligibility for amyloid-lowering DMTs, panel f – quadratic-weighted *κ̂*_G2_ for microhemorrhages estimation. A, B, and C are neuroradiologists. D is the neurologist. Percent agreement is shown in italics, *κ̂*_G_ coefficient is shown in bold with 95% CI in parenthesis.

Abbreviations: CI, confidence interval; DMTs, disease modifying therapies; MTA, medial temporal lobe atrophy
